# 2′-Fucosyllactose mitigates cognitive deficits in Alzheimer models: targeting amyloid pathology, oxidative stress, and synaptic plasticity

**DOI:** 10.3389/fphar.2025.1598030

**Published:** 2025-08-21

**Authors:** Yeasmin Akter Munni, Khoa Nguyen Tran, Seon-Min Jeon, Meeyul Hwang, Jong-Won Yoon, Young-Ha Song, Tae Woo Oh, Sang Il Gum, In-Jun Yang

**Affiliations:** ^1^ Department of Physiology, Dongguk University College of Korean Medicine, Gyeongju, Republic of Korea; ^2^ QBGEN Inc., Gyeongsan, Republic of Korea; ^3^ Advanced Protein Technologies Corp., Suwon, Republic of Korea; ^4^ Korean Medicine (KM)-Application Center, Korea Institute of Oriental Medicine (KIOM), Daegu, Republic of Korea; ^5^ Department of Korean Convergence Medical Science, University of Science & Technology (UST), Daejeon, Republic of Korea; ^6^ Reverse Aging Holdings Co., Ltd., Seoul, Republic of Korea

**Keywords:** 2′-fucosyllactose, Alzheimer’s disease, cognitive function, oxidative stress, synaptic plasticity, 5xFAD, d-galactose

## Abstract

**Introduction:**

The development of new drugs for Alzheimer’s disease (AD) remains a major challenge due to the disorder’s complex and multifactorial nature. 2′-Fucosyllactose (2′-FL), a human milk oligosaccharide, has demonstrated promising neuroprotective properties. However, its effects on AD-related cognitive decline are not yet fully understood. This study aimed to investigate the therapeutic potential of 2′-FL in an aging mouse model of AD and to explore the underlying mechanisms involved.

**Methods:**

5xFAD transgenic mice were treated with 2′-FL and assessed for cognitive function using the Morris water maze and Y-maze tests. Immunohistochemical staining was used to evaluate amyloid-beta (Aβ) and phosphorylated tau (p-tau) levels in brain tissue samples. Blood samples were analyzed to determine circulating cytokine levels. Additionally, BV2 microglial cells and primary hippocampal neurons (PHNs) were used *in vitro* to investigate the effects of 2′-FL on neuroinflammation, oxidative stress, and synaptic plasticity.

**Results:**

2′-FL (300–1,200 mg/kg, oral) improved cognitive performance in 5xFAD mice by shortening escape latency in the water maze and restoring alternation behavior in the Y-maze test. It significantly reduced Aβ plaque load in the hippocampus and cortex but did not significantly affect tau hyperphosphorylation. Furthermore, 2′-FL lowered plasma tumor necrosis factor (TNF)-α and interleukin (IL)-6 levels. In BV2 cells, it suppressed d-galactose-induced neuroinflammation by downregulating TNF-α and IL-6, and nuclear factor-κB signaling. In PHNs, 2′-FL reduced oxidative stress, restored mitochondrial function, and limited DNA damage. Additionally, it counteracted d-galactose-induced synaptic deficits by promoting neurite outgrowth, enhancing synaptic vesicle recycling, and upregulating the synaptic markers brain-derived neurotrophic factor, postsynaptic density protein-95, and synaptic vesicle protein 2.

**Conclusion:**

2′-FL improved cognitive performance in 5xFAD mice, reduced Aβ plaque deposition and pro-inflammatory cytokine levels *in vivo*, and mitigated oxidative stress and synaptic dysfunction in cellular models. These findings indicate that 2′-FL modulates multiple pathological features relevant to AD in preclinical models.

## Graphical Abstract

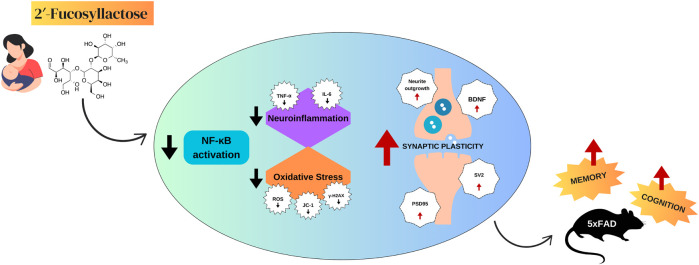

## 1 Introduction

Alzheimer’s disease (AD) is a progressive neurodegenerative disorder affecting over 55 million people worldwide, with its prevalence and healthcare burden expected to rise significantly in the coming decades (Alzint, 2025). Despite extensive research, effective disease-modifying therapies remain scarce, largely because of the complex and multifactorial nature of AD pathology (Dissanayaka et al., 2024). At the core of AD is a vicious cycle involving amyloid-beta (Aβ) accumulation, tau hyperphosphorylation, oxidative stress, neuroinflammation, and synaptic dysfunction. Aβ aggregates and hyperphosphorylated tau disrupt neuronal function and impair mitochondrial activity, leading to excess reactive oxygen species (ROS) production (Zhang et al., 2022; Choi et al., 2023). This oxidative stress damages lipids, proteins, and nucleic acids, further compromising neuronal integrity and synaptic signaling. In turn, injured neurons trigger activation of microglia and astrocytes, amplifying neuroinflammation (Plascencia-Villa and Perry, 2023). The inflammatory cascade promotes further Aβ deposition and tau pathology, creating a self-perpetuating cycle that accelerates cognitive decline. These interlinked mechanisms underscore the need for therapies that can simultaneously target multiple aspects of AD progression (Choi et al., 2023).

Recent advancements in AD treatment have introduced several disease-modifying therapies targeting core pathological mechanisms. Monoclonal antibodies like lecanemab and donanemab, approved by the FDA for early-stage AD, work by binding to and promoting the clearance of Aβ plaques. While promising, these therapies are costly, require regular intravenous administration, and carry risks of amyloid-related imaging abnormalities, such as cerebral edema and hemorrhage (Espay et al., 2024; Hartz et al., 2025). Traditional drugs, including cholinesterase inhibitors (e.g., donepezil, rivastigmine) and the NMDA receptor antagonist memantine, remain in use for symptomatic relief but do not alter disease progression and can cause significant side effects (Alzheimer’s Association, 2025). Emerging therapies are also exploring non-amyloid targets. For example, Xanamem, which inhibits cortisol production, offers a novel approach distinct from conventional strategies (Villemagne et al., 2024). These developments reflect a growing shift toward multifaceted treatment strategies addressing the complex pathology of AD.

2′-Fucosyllactose (2′-FL), a major human milk oligosaccharide, has gained attention for its neuroprotective potential. Preclinical studies show that oral 2′-FL enhances hippocampal long-term potentiation and cognitive performance in rodents, accompanied by increased expression of synaptic proteins such as PSD-95, p-CaMKII, and BDNF (Vázquez et al., 2015). It also reduces oxidative stress and apoptosis in neuronal cultures, and improves cognitive outcomes in d-galactose-induced AD models by lowering oxidative stress markers, boosting antioxidant enzymes, and modulating gut microbiota composition (Wang et al., 2022; Gao et al., 2024). In a stroke model, 2′-FL reduced neurodegeneration, suppressed microglial activation, and upregulated BDNF expression, further supporting its neuroprotective role (Wu et al., 2020).

Despite these promising findings, many implicating the gut-brain axis, the broader impact of 2′-FL on AD pathology remains unclear. Therefore, this study aimed to investigate the multifaceted effects of 2′-FL using the 5xFAD transgenic mouse model of AD, alongside two *in vitro* models: d-galactose-treated BV2 microglial cells and primary hippocampal neurons (PHNs). Through behavioral testing and molecular analysis, we sought to clarify the mechanisms by which 2′-FL may offer neuroprotection in the context of AD.

## 2 Materials and methods

### 2.1 *In vivo* experiments

#### 2.1.1 Animal grouping and treatment

**FIGURE 1:**
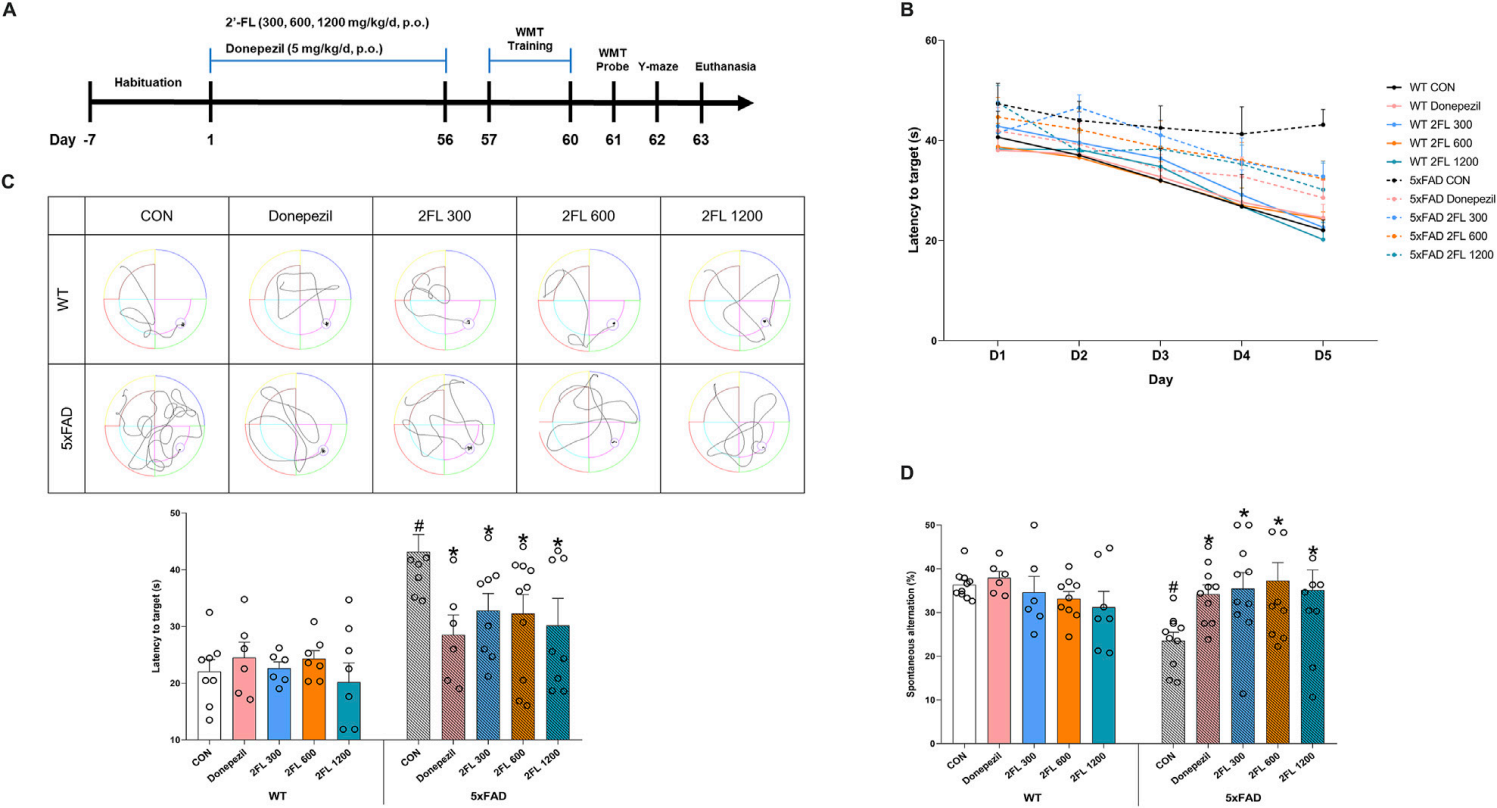
Effect of 2′-FL on mouse memory and cognition. **(A)** Schematic timeline of *in vivo* experiments. **(B)** Latency to reach the platform (zone) during the training period (day 1 to day 4) and probe test (day 5) in the WMT. **(C)** Latency to reach the zone of platform in day 5. **(D)** Percentage of spontaneous alternation in the Y-maze test. Data are presented as mean ± SEM (n = 6-10 per group). #p < 0.05 vs. WT CON, *p < 0.05 vs. 5xFAD CON (One-way ANOVA followed by Holm-Sidak’s multiple comparisons test).

The animal study was conducted in accordance with the institutional guidelines outlined by the Principles of Laboratory Animal Care (NIH publications No. 85-23, revised 1996) and the Korea Institute of Oriental Medicine Institutional Animal Care and Use Committee (KIOM-IACUC, reference number 24–073). Twenty-four-week-old female 5xFAD transgenic mice and age-matched wild-type C57BL/6 mice were obtained from The Jackson Laboratory (Bar Harbor, ME, United States). Mice were randomly group-housed (n = 10 per group) with free access to food (Purina Korea, Seoul, Korea) and water. Mice were kept under pathogen-free conditions at a temperature of 23 °C ± 1 °C on an alternating 12-h light and dark cycle. 2′-FL (Fuc-α1,2-Gal-β1,4-Glc; Advanced Protein Technologies Corp., Suwon, Republic of Korea) was administered orally at doses of 300, 600, and 1,200 mg/kg/day for 8 weeks through a gastric tube. The dose of 2′-FL was selected based on two previous studies, with the lowest dose (300 mg/kg/day) approximating the average amount of 2′-FL that breast-fed infants would receive (Vazquez et al., 2016; Gao et al., 2024). The positive control group was orally administered donepezil 5 mg/kg/day. Distilled water (DW) was administered to the control animals using the same feeding methods over the same period. The schematic timeline of this experiment is shown in Figure 1A.

#### 2.1.2 Water maze test (WMT)

A circular water tank (diameter, 150 cm; height, 60 cm) was filled with water to a depth of 30 cm, with an escape platform placed in one quadrant. During a 4-day training period, the mice were allowed to swim freely for 60 s without the platform to acclimatize to the environment. They then underwent four training sessions per day, with the entry point varying across trials. The escape platform, submerged 1 cm below the surface, was invisible during testing. All sessions were recorded using a video tracking system (SMART v3.0, Panlab SL, Barcelona, Spain). If a mouse located the platform within 60 s, it remained there for 15 s; otherwise, it was gently guided to the platform and allowed to stay for 20 s. During the probe test, the escape platform was removed, and the time spent in the target zone, where the platform had previously been located, was recorded over a 60-s period.

#### 2.1.3 Y-maze test

The Y-maze consisted of three arms (42 cm long, 3 cm wide, 12 cm high) arranged at 120° angles and constructed using black polyvinyl plastic. The arms were randomly labeled A, B, and C. Each mouse was placed in one arm and allowed to explore freely for 8 min. Arm entries were recorded, with a valid entry defined as the full entry of the mouse’s tail into an arm. Reentries into the same arm were also noted. Spontaneous alternation behavior was defined as successive entries into all three arms in a triplet sequence.

### 2.2 *In vitro* experiments

#### 2.2.1 BV2 cell culture and treatment

BV2 microglia were cultured in Dulbecco’s modified Eagle medium (DMEM) supplemented with 10% FBS (Merck KGaA, Darmstadt, Germany) and 1% penicillin-streptomycin (Thermo Fisher Scientific, Waltham, MA, United States). Water-soluble tetrazolium salt assay was performed to screen the non-toxic dose of 2′-FL on the cell line. Cells were seeded in 96-well plates (3 × 10^4^ cells/well) and incubated at 37 °C and 5% CO2 for 24 h. Cells were treated with 2′-FL (0.5–8 mg/mL) or DW for 24 h, followed by quantification of viability levels through absorbance at 450 nm (detection wavelength) and 650 nm (reference wavelength) using a Tecan microplate reader (Männedorf, Switzerland).

#### 2.2.2 Primary hippocampal neuronal cell culture and treatment

All animal experiments were conducted with the approval of the Institutional Animal Care and Use Committee of Dongguk University College of Medicine (IACUC-2021-13). Pregnant E−16 female Sprague–Dawley rats were purchased from Orient Bio Inc. (Seongnam-si, Korea), maintained for 3 days with free access to food and water at room temperature (RT) under a 12-h light/dark cycle, and euthanized via cervical dislocation. The brains were harvested in precooled Hanks’ balanced salt solution (PAA, Pasching, Austria), hippocampi were meticulously dissected, and cell suspensions were prepared following a previously established methodology (Munni et al., 2023a; Munni et al., 2023b). A specific serum-free neurobasal medium supplemented with B27 was added to 24-well plates containing 12-mm coverslips coated with poly-d-lysine (PDL). Cells were seeded onto coverslips at a density of 3 × 10^4^ cells/cm^2^ for morphometric analysis, cell viability, and immunocytochemistry to determine the required protein expression, and at a density of 6 × 10^4^ cells/cm^2^ for synaptogenesis. The plates were then incubated at 37 °C with 5% CO2 and 95% air for 3 days *in vitro* (DIV3), DIV8, and DIV9. 2′-FL (0.5, 1, and 2 mg/mL), d-galactose (50 mg/mL), or vehicles were added into the culture media prior to cell seeding. Fresh medium with treatment was given to the cells in a 4-day cycle. The schematic timeline of this experiment is shown in Figure 4A.

### 2.3 Biochemical and molecular assays

#### 2.3.1 Thioflavin S staining

The brain was dissected from perfused mice and post-fixed in 4% paraformaldehyde (PFA) for 8 h at 4 °C. Tissues were subsequently immersed in 30% sucrose at 4 °C until fully submerged. Coronal brain sections were prepared using a brain matrix, and tissue samples were transferred into tissue clearing solution (HRTC-001, Binaree, Daegu, Korea) and incubated at 38 °C on an orbital shaker. Incubation times varied depending on the organ (24 h for 1 mm brain sections). For visualization of amyloid beta plaques, samples were treated with thioflavin S, which emits fluorescence at 488 nm. Plaque staining was performed using 0.1% thioflavin S in 50% ethanol, incubated at 25 °C with gentle shaking at 50 rpm for 15 min, followed by three washes with 1× PBS at 4 °C for 20 min each. Samples were then incubated in 10 mL of mounting and storage solution (HRMO, Binaree, Daegu, Korea) at 38 °C for 24 h with shaking at 50 rpm to match the refractive index of the tissue. Imaging was conducted using a confocal microscope to detect thioflavin S fluorescence in the green channel. Image acquisition and analysis were performed using the Imaris software (version 9.5.1, Oxford Instruments, Abingdon-on-Thames, United Kingdom).

#### 2.3.2 Immunostaining

For immunofluorescence staining, brain tissue sections were washed with 1× PBS and then sectioned into 0.1-mm-thick sections using a vibratome (Vibratome VT1200, Leica, Wetzlar, Germany). Subsequently, the tissues were stained using Binaree Tissue Clearing for Immunostaining (HRTI-001, Amuza Inc., San Diego, CA, United States) to allow easy antibody penetration into the thick tissue. Briefly, paraformaldehyde-fixed brains were immersed in fixing solution at 4 °C overnight and incubated with tissue clearing solutions A and B at 37 °C for 24 h in a shaking incubator. Primary antibodies and concentrations were anti-beta amyloid (1:100, Abcam, Cambridge, United Kingdom), anti-phospho tau antibody (1:100, Swant, Bellinzona, Switzerland). The secondary antibody used for immunofluorescence detection was Alexa Fluor 488 AffiniPure F (ab’)2 Fregment Goat Anti-Rabbit IgG (H + L) (1:200; Jackson ImmunoResearch Laboratories, Inc.). The sliced brains were then incubated in mounting and storage solution (HRMO, Binaree, Daegu, Korea) for 24 h to match the refractive index.

#### 2.3.3 ELISA

For *in vivo* analysis, blood was collected from the retro-orbital plexus of experimental mice using EDTA-treated Pasteur pipettes. Samples were centrifuged, and plasma was separated and stored at −20 °C until analysis. Cytokine levels, including tumor necrosis factor-alpha (TNF-α) and interleukin-6 (IL-6), were measured using mouse ELISA kits (Endogen Inc., Cambridge, MA, United States) according to the manufacturer’s instructions. For the *in vitro* analysis, BV2 cells were seeded at 2 × 10^5^ cells/well in 12-well plates and incubated for 24 h. Cells were then treated with 2′-FL for 5 h, followed by d-galactose (10 mg/mL) for an additional 22 h, based on a previously established protocol. Culture media were collected, centrifuged at 1,500 rpm for 10 min at 4 °C, and the supernatants were analyzed for TNF-α and IL-6 levels using ELISA kits (LABISKOMA, Seoul, South Korea). Absorbance was measured at 450 nm using a microplate reader (Tecan, Männedorf, Switzerland).

#### 2.3.4 Western blot

BV2 cells were seeded at a density of 3 × 10^6^ cells/100-mm dish and incubated for 24 h. Cells were then treated with 2′-FL for 5 h followed by d-galactose (10 mg/mL) for another 30 min. Cytoplasmic and nuclear fractions were extracted using NE-PER™ Nuclear and Cytoplasmic Extraction Reagents (Thermo Fisher Scientific), following the manufacturer’s protocol. Prepared samples (30 μg/lane) were separated by SDS-PAGE using a 5% stacking gel and a 10% resolving gel. Proteins were then transferred onto polyvinylidene fluoride membranes (Merck Millipore, Carrigtwohill, Ireland). Membranes were blocked with 5% skim milk for 2 h at RT then incubated overnight at 4 °C with primary antibodies, followed by a 1-h incubation with HRP-conjugated secondary antibodies at RT. After washing, immunoreactive bands were visualized using ClarityTM Western ECL substrate (Bio-Rad, Hercules, CA, United States), and images were captured using a ChemiDoc MP Imaging System (Bio-Rad). Densitometric analysis was performed using the Gel-Pro Analyzer version 3.1 software (Media Cybernetics, Rockville, MD, United States). Primary antibodies included rabbit anti-IκBα (Cell Signaling, Danvers, MA, United States), rabbit anti-NF-κB (Cell Signaling), mouse anti-β-actin (Sigma-Aldrich, St. Louis, MO, United States), mouse anti-PCNA (Santa Cruz Biotechnology, Dallas, TX, United States), anti-rabbit IgG HRP-linked whole antibody (Amersham ECL, Little Chalfont, United Kingdom), and goat anti-mouse IgG (Enzo Life Sciences, Farmingdale, NY, United States).

#### 2.3.5 Immunocytochemistry

Neurons on the coverslips were rinsed twice with 1x Dulbecco’s PBS (D-PBS; Invitrogen) and fixed using a progressive fixation process with 4% paraformaldehyde and 10% methanol. Cells were permeabilized with 0.1% Triton X-100 and blocked with 2.5% goat serum in 0.2% Tween-20 in PBS, followed by two washes with 1×PBS. Subsequently, neurons were incubated overnight at 4 °C with the following primary antibodies: rabbit anti-BDNF (Abclonal, Woburn, MA, United States), mouse anti-phospho-H2AX (Millipore, Billerica, MA, United States), mouse anti-tubulin α (Developmental Studies Hybridoma Bank, University of Iowa, Iowa City, IA, United States), rabbit anti-GABAB receptor 1 (Abclonal), and mouse anti-glutamic acid decarboxylase (Developmental Studies Hybridoma Bank, University of Iowa). After washing five times for 5 min, the cells were treated with Alexa Fluor 488/568-conjugated goat anti-mouse/rabbit IgG (Invitrogen, Carlsbad, CA, United States) for 2.5 h. A mounting solution containing DAPI was used to secure the coverslips onto the glass slides.

#### 2.3.6 Primary hippocampal neuron (PHN) viability

Neuronal viability was assessed using the trypan blue exclusion assay. On DIV3, neurons were incubated with 0.4% (w/v) trypan blue for 25 min at 37 °C, then rinsed with 1× D-PBS. Cells were observed under a light microscope, and viability was calculated as the percentage of unstained (viable) cells relative to the total number of cells. Non-viable neurons, which take up the dye due to compromised membrane integrity, appeared dark blue, while viable neurons excluded the dye and remained clear.

#### 2.3.7 Dichlorohydrofluorescein diacetate (DCFHDA) dye staining

ROS generation was assessed using DCFH-DA (MedChemExpress, Monmouth Junction, NJ, United States). Neurons were seeded on PDL-coated coverslips at a density of 3 × 10^4^ cells/cm^2^ in 24-well plates. On DIV3, following d-galactose treatment, cells were incubated with 5 μM DCFH-DA for 30 min at 37 °C. After incubation, the cells were washed twice with 1 × DPBS and imaged using a fluorescence microscope at 488 nm. ROS levels were quantified by measuring DCF fluorescence intensity using ImageJ software (version 1.49; NIH, Bethesda, MD, United States).

#### 2.3.8 JC-1 staining

Neurons were seeded on PDL-coated coverslips in 24-well plates at a density of 3 × 10^4^ cells/cm^2^. On DIV3, after d-galactose treatment, cells were incubated with 5,5′,6,6′-tetrachloro-1,1′,3,3′-tetraethylbenzimidazolyl-carbocyanine iodide (JC-1) (MedChemExpress) at a concentration of 5 μg/mL for 30 min at 37 °C, then washed twice with 1 × DPBS. Fluorescence images were captured to detect JC-1 monomers (green, 488 nm) and aggregates (red, 568 nm). Mitochondrial membrane potential (MMP) was quantified by calculating the red-to-green fluorescence ratio using ImageJ software (version 1.49).

#### 2.3.9 FM1-43 staining

Neurons were cultured on PDL-coated coverslips (3 × 10^4^ cells/cm^2^) until DIV8 and then treated with d-galactose for 24 h. After treatment, the cells were washed with 1 × DPBS and incubated with N-(3-triethylammoniumpropyl)-4-(4-(dibutylamino)styryl) pyridinium dibromide (FM1-43) (Invitrogen) at a final concentration of 5 μg/mL for 3 min. FM1-43 is rapidly taken up by recycling synaptic vesicles, labeling active nerve terminals. Cells were rinsed twice with 1× D-PBS to remove nonspecific membrane staining, and synaptic vesicle (SV) puncta were visualized using a fluorescence microscope (excitation at 568 nm). Fluorescence intensity of SV puncta was quantified using ImageJ software (version 1.49).

#### 2.3.10 Image acquisition

A fluorescence microscope (DFC3000G, Leica) equipped with a 1.3-megapixel Sony^®^ (Tokyo, Japan) CCD monochrome sensor P and Leica Application SuiteX (LasX Version 3.7.2.22383) software was used to monitor and photograph the cells. Adobe Photoshop 2020 (Adobe, San Jose, CA, United States) was used for image processing. Morphometric analysis was performed using ImageJ (version 1.49; NIH) to quantify the number and length of primary neurites, the length of the longest neurite, and the number of synaptic puncta per neuron. For synaptic puncta analysis and visualization of synaptic plasticity-related protein expression, images were acquired using the Cellsens software (version 1.18; Olympus, Tokyo, Japan) with an Olympus BX53 microscope equipped with a DP74 1/1.2-inch Color CMOS camera.

### 2.4 Statistical analysis

Statistical analyses were performed using GraphPad Prism version 8.0.1 (GraphPad Software, San Diego, CA, United States). The Shapiro-Wilk test assessed data normality, while the Brown-Forsythe test checked for equal variances. For data that met assumptions of normality and equal variance, one-way ANOVA followed by Holm-Sidak’s *post hoc* test was used. If the data were normally distributed but showed unequal variances, Welch’s ANOVA with Dunnett’s T3 *post hoc* test was employed. Non-normally distributed datasets were analyzed using the Kruskal–Wallis test and Dunn’s *post hoc* comparison. Data are expressed as the mean ± standard error of the mean (SEM), with statistical significance set at p < 0.05.

## 3 Results

### 3.1 2′-FL enhances memory function in 5xFAD mice

To assess the effects of 2′-FL on cognitive function, we conducted behavioral tests in wild-type (WT) and 5xFAD mice. During the WMT training phase, escape latency decreased in all groups except the 5xFAD control (CON) group, indicating impaired spatial learning in these mice (Figure 1B). In the probe test, the 5xFAD CON group (43.14 ± 3.07 s) showed a significantly longer latency to the target zone compared to the WT CON group (22.06 ± 2.09 s). This impairment was significantly reversed by 2′-FL at all tested doses (300 mg/kg, 32.80 ± 3.02; 600 mg/kg, 32.26 ± 3.36; and 1,200 mg/kg, 30.15 ± 4.79 s) and by donepezil (28.54 ± 3.49 s) (p < 0.05, Figure 1C). Similarly, in the Y-maze test, 5xFAD CON (23.52 ± 1.96 s) mice exhibited a significantly lower spontaneous alteration percentage compared to WT CON (36.34 ± 1.05 s). This reduction was significantly ameliorated by 2′-FL (300 mg/kg, 35.46 ± 3.66; 600 mg/kg, 37.29 ± 4.14; and 1,200 mg/kg, 35.11 ± 4.61 s) and donepezil (34.20 ± 2.10 s) (p < 0.05, Figure 1D). These findings indicate that 2′-FL effectively improves cognitive performance *in vivo*.

### 3.2 2′-FL suppresses Aβ accumulation in both the hippocampus (HPC) and cortex of 5xFAD mice

**FIGURE 2:**
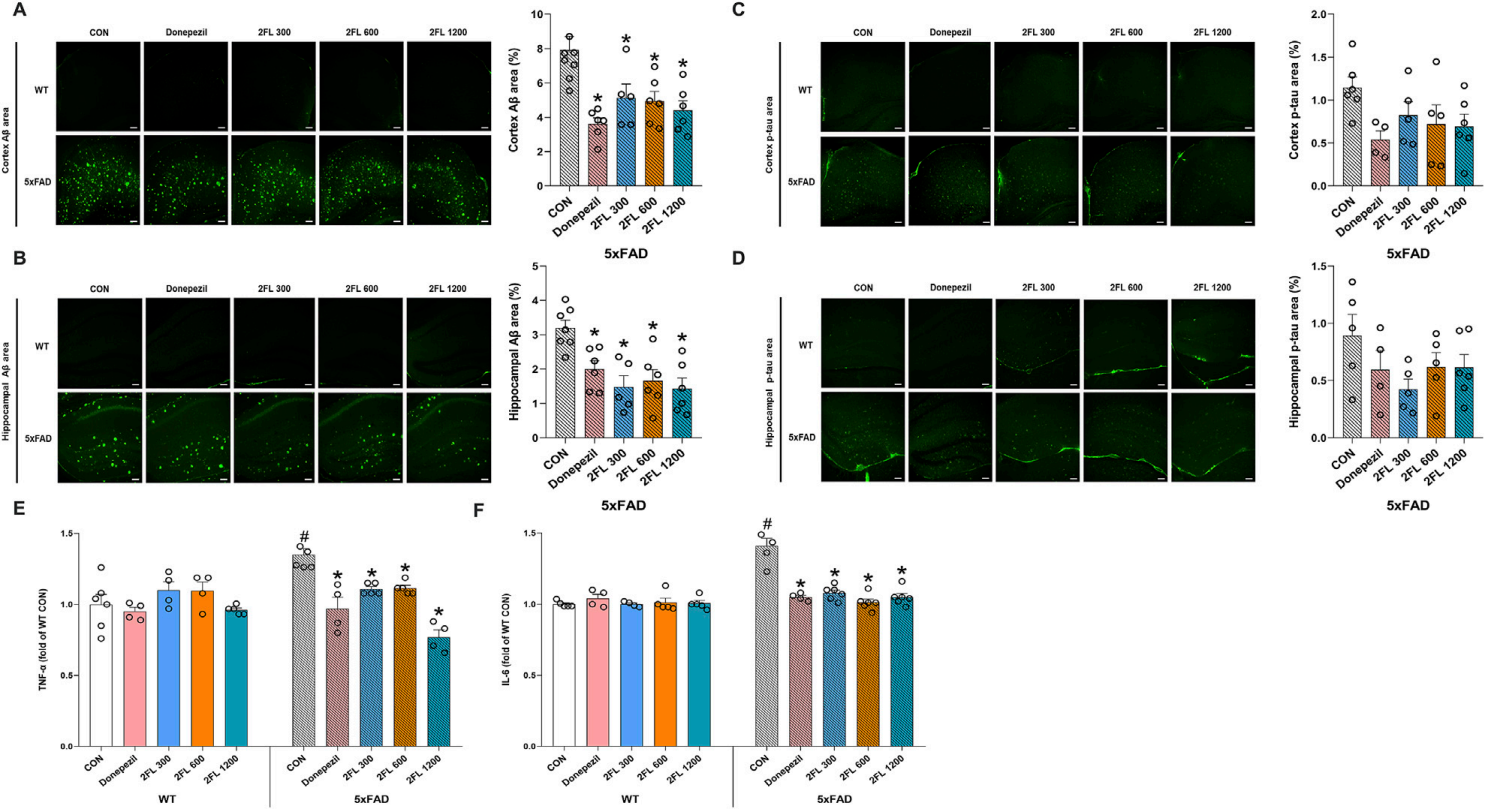
Effect of 2′-FL on molecular markers *in vivo*. Expression of Aβ accumulation in the **(A)** cortex and **(B)** HPC of 5xFAD mice. Expression of p-tau in the **(C)** cortex and **(D)** HPC of 5xFAD mice. Levels of plasma **(E)** TNF-α and **(F)** IL-6 in WT and 5xFAD mice. Data are presented as mean ± SEM (n = 4-6 per group). #p < 0.05 vs. WT CON, *p < 0.05 vs. 5xFAD CON (Panels A–E: One-way ANOVA followed by Holm-Sidak’s multiple comparisons test; Panel F: Welch’s ANOVA with Dunnett’s T3 multiple comparisons test).

To evaluate the effect of 2′-FL on Aβ pathology, we performed immunohistochemical staining and quantification of Aβ- and p-tau-positive areas in the HPC and cortex. The 5xFAD CON mice (cortex, 7.91 ± 0.79; HPC, 3.19 ± 0.22%) exhibited a high accumulation of Aβ plaques in both regions, and treatment with donepezil (cortex, 3.61 ± 0.35; HPC, 1.99 ± 0.24%) and 2′-FL at 300, 600, and 1,200 mg/kg (cortex: 300 mg/kg, 5.13 ± 0.80; 600 mg/kg, 4.94 ± 0.56; and 1,200 mg/kg, 4.41 ± 0.55; HPC: 300 mg/kg, 1.47 ± 0.32; 600 mg/kg, 1.65 ± 0.32; and 1,200 mg/kg, 1.42 ± 0.30%) significantly reduced Aβ deposition (p < 0.05, Figures 2A,B). The expression of p-tau in the cortex and HPC was higher in 5xFAD CON mice (cortex, 1.14 ± 0.12; HPC: 0.89 ± 0.18%) than in normal control mice, and there was a general decrease in both brain regions in all 2′-FL-treated groups (cortex: 300 mg/kg, 0.82 ± 0.15; 600 mg/kg, 0.71 ± 0.22; and 1,200 mg/kg, 0.69 ± 0.14; HPC: 300 mg/kg, 0.42 ± 0.08; 600 mg/kg, 0.61 ± 0.12; and 1,200 mg/kg, 0.61 ± 0.11%); however, these changes were not significant (p > 0.05, Figures 2C,D). These results indicate that while 2′-FL exerts a robust effect on Aβ pathology, its impact on tau hyperphosphorylation appears limited.

Next, to investigate whether 2′-FL mitigates inflammation *in vivo*, we quantified the plasma levels of the pro-inflammatory cytokines TNF-α and IL-6 across experimental groups. Compared to the WT CON group (TNF-α, 1.00 ± 0.07; IL-6: 1.00 ± 0.01), vehicle-treated 5xFAD mice (TNF-α, 1.35 ± 0.04; IL-6: 1.41 ± 0.05) showed remarkably higher TNF-α and IL-6 levels (p < 0.05), whereas those of 5xFAD mice receiving donepezil (TNF-α, 0.97 ± 0.08; IL-6: 1.05 ± 0.01) and 2′-FL (TNF-α: 300 mg/kg, 1.11 ± 0.02; 600 mg/kg, 1.12 ± 0.02; and 1,200 mg/kg, 0.77 ± 0.05; IL-6: 300 mg/kg, 1.08 ± 0.02; 600 mg/kg, 1.01 ± 0.02; and 1,200 mg/kg, 1.05 ± 0.02%) declined significantly (p < 0.05, Figures 2E,F).

### 3.3 2′-FL alleviated d-galactose-induced neuroinflammation in BV2 cells

**FIGURE 3:**
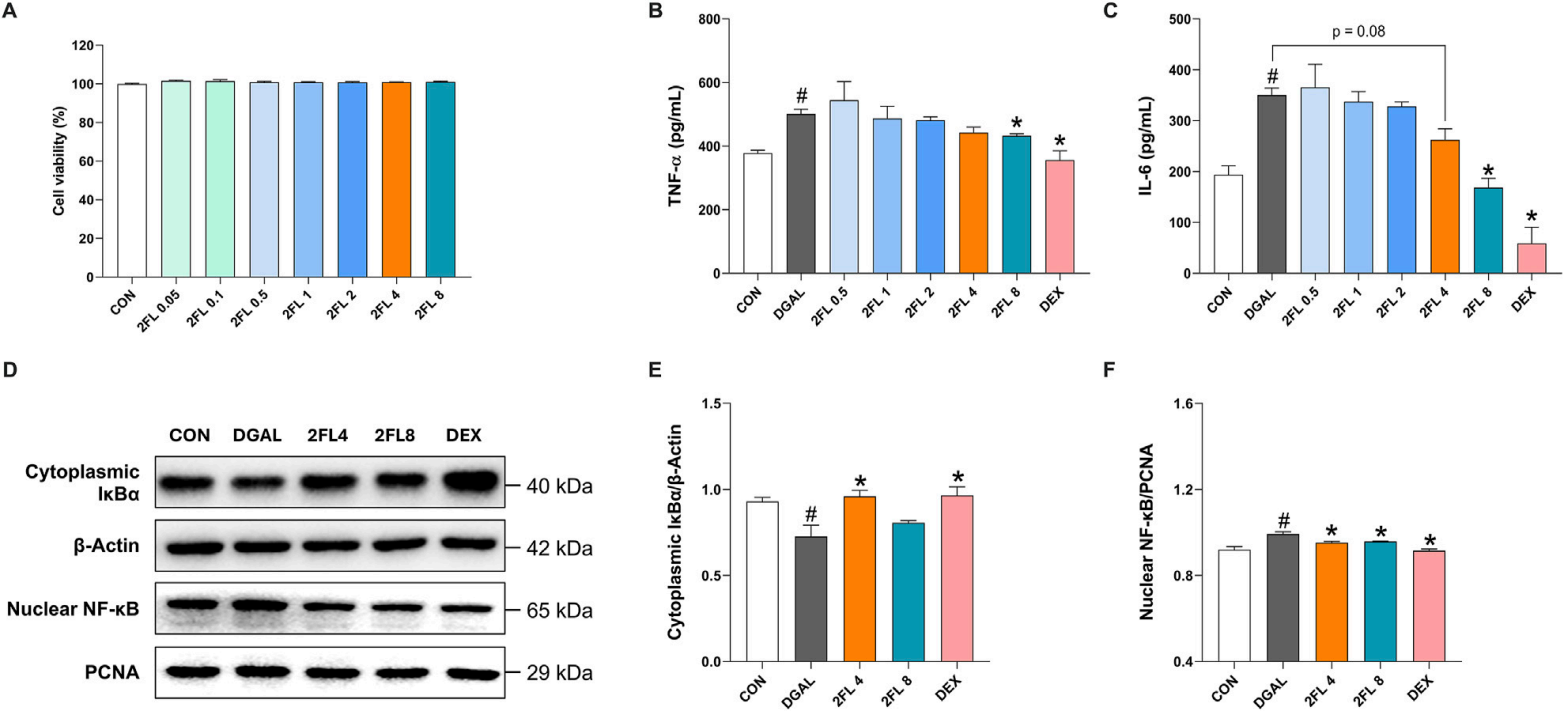
Effect of 2′-FL on D-galactose-induced neuroinflammation in BV2 cells. **(A)** Cell viability under 2′-FL treatments. Quantification of **(B)** TNF-α and **(C)** IL-6 levels in the cell culture media. **(D–F)** Protein expression of IκBα (cytoplasmic fraction) and NF-κB (nuclear fraction) in d-galactose-treated cells. Data are presented as mean ± SEM (n = 3 per group). #p < 0.05 vs. CON, *p < 0.05 vs. DGAL (One-way ANOVA followed by Holm-Sidak’s multiple comparisons test).

To further investigate the anti-neuroinflammatory properties of 2′-FL, its effects on d-galactose-treated BV2 cells were studied. The d-galactose dose (10 mg/mL) was selected based on a previous report (Fan et al., 2023). D-galactose at 10 mg/mL significantly increased TNF-α and IL-6 levels in BV2 culture media (TNF-α, 501.3 ± 14.73; IL-6: 350.6 ± 13.83 pg/mL). Regarding treatments with 2′-FL from 0.5 to 8 mg/mL, cytokine levels tended to decrease in a dose-dependent manner, but only 8 mg/mL 2′-FL exerted significant effects (TNF-α, 432.3 ± 6.34; IL-6: 168.4 ± 18.48 pg/mL) (p < 0.05, Figures 3A–C). Western blot analysis demonstrated that d-galactose was associated with reduced cytoplasmic IκBα (0.72 ± 0.06) and increased nuclear NF-κB (0.99 ± 0.01) levels, indicating NF-κB activation. However, treatments with the positive control DEX (IκBα, 0.96 ± 0.04; NF-κB, 0.99 ± 0.01) and 2′-FL 4 μg/mL (IκBα, 0.96 ± 0.04; NF-κB, 0.95 ± 0.00) counteracted these effects (p < 0.05, Figures 3D–F). Collectively, these findings suggest that 2′-FL mitigates neuroinflammation by suppressing proinflammatory cytokine expression and inhibiting NF-κB signaling in microglia.

### 3.4 2′-FL improved d-galactose-induced oxidative stress in PHNs

To determine the optimal dose of d-galactose, a 0.4% trypan blue exclusion assay was conducted. Treatment with d-galactose at concentrations of 5–25 mg/mL reduced cell viability to 80%–90% compared with the control group, whereas 50 mg/mL resulted in a more pronounced decline to 66%. Representative bright-field images revealed dead cells, indicated by yellow arrowheads, alongside marked inhibition of neurite outgrowth (Supplementary Figure S1). Based on these findings, 50 mg/mL d-galactose was selected for subsequent experiments.

**FIGURE 4:**
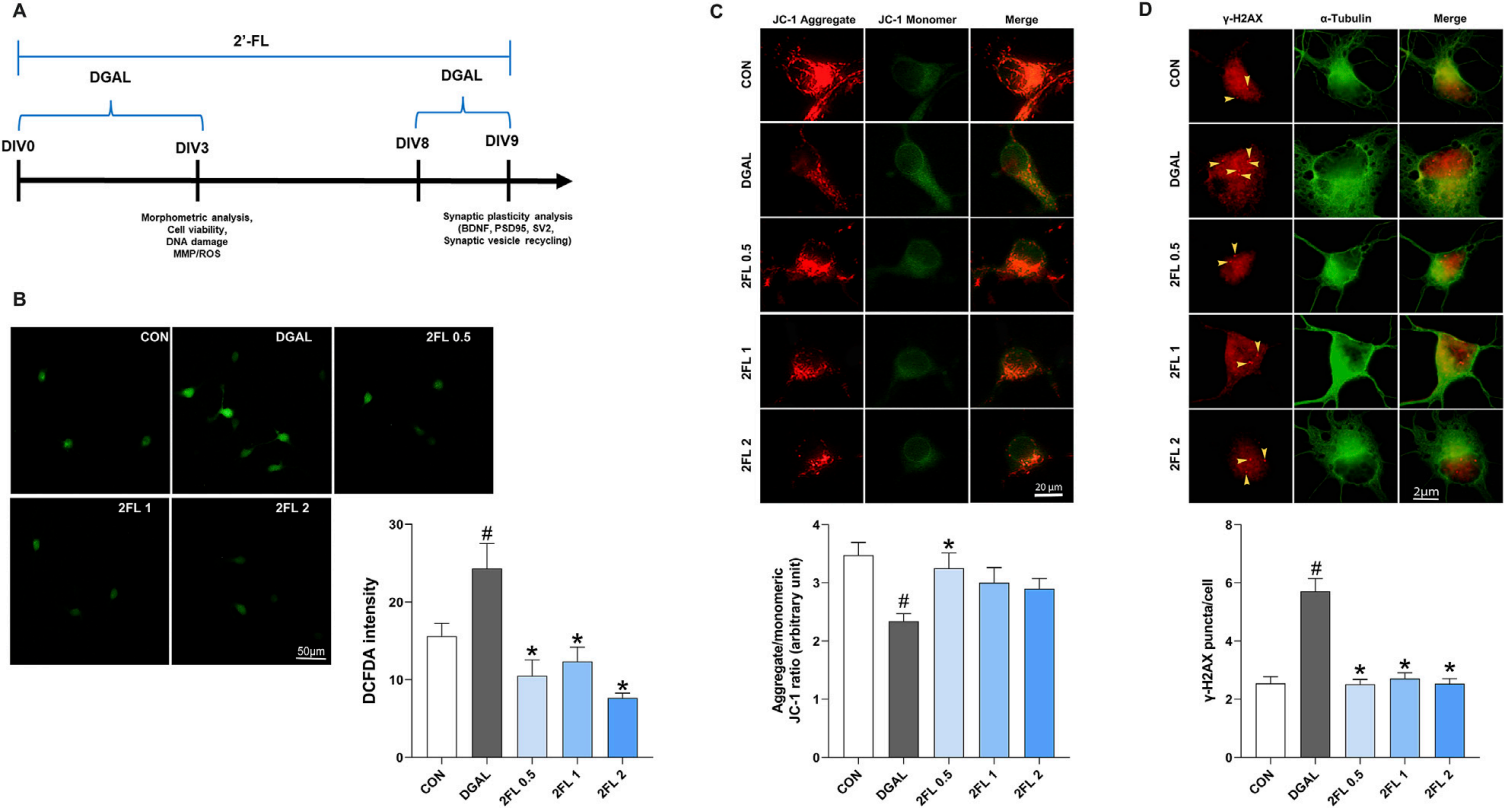
Effect of 2′-FL on D-gal-induced oxidative stress in hippocampal primary neurons. **(A)** Schematic timeline of *in vitro* experiments in primary neurons. Fluorescence intensity of **(B)** ROS stained with DCFDA**, (C)** JC-1 aggregate, and **(D)** γ-H2AX puncta. Data are presented as mean ± SEM (30 neurons per group, and each experiment was independently repeated three times). #p < 0.05 vs. CON, *p < 0.05 vs. DGAL (Panel B: One-way ANOVA followed by Holm-Sidak’s multiple comparisons test; Panels C and D: Kruskal–Wallis with Dunn’s multiple comparisons test).

To evaluate the potential of 2′-FL to mitigate d-galactose-induced oxidative stress and mitochondrial dysfunction, we evaluated ROS levels and MMP after 72 h of d-galactose treatment. First, PHNs treated with d-galactose (24.32 ± 3.22) exhibited a significant increase in DCFH-DA fluorescence intensity compared with control neurons (15.60 ± 1.66), indicating elevated ROS levels (p < 0.05, Figure 4B). Treatment with 2′-FL at 0.5, 1, and 2 mg/mL (0.5 μg/mL, 10.49 ± 2.06; 1 μg/mL, 12.36 ± 1.84; 2 μg/mL, 7.65 ± 0.61) significantly reduced ROS accumulation (p < 0.05, Figure 4B). JC-1 staining revealed that mitochondria in the d-galactose-treated group (2.34 ± 0.12) predominantly emitted green fluorescence, reflecting MMP loss (p < 0.05, Figure 4C). Conversely, 2′-FL 0.5 μg/mL (3.25 ± 0.26) resulted in a shift toward red fluorescence, indicating the recovery of MMP (p < 0.05, Figure 4C). Lastly, to assess DNA damage, cells were immunostained for phosphorylated γ-H2AX and α-tubulin. Compared to the d-galactose-treated group (5.71 ± 0.44), neurons treated with 2′-FL (0.5 μg/mL, 2.51 ± 0.16; 1 μg/mL, 2.71 ± 0.19; 2 μg/mL, 2.53 ± 0.17) exhibited a marked reduction in γ-H2AX foci, indicating a decrease in DNA damage (p < 0.05, Figure 4D).

### 3.5 2′-FL reversed the effects of d-galactose on synaptic plasticity in PHNs

**FIGURE 5:**
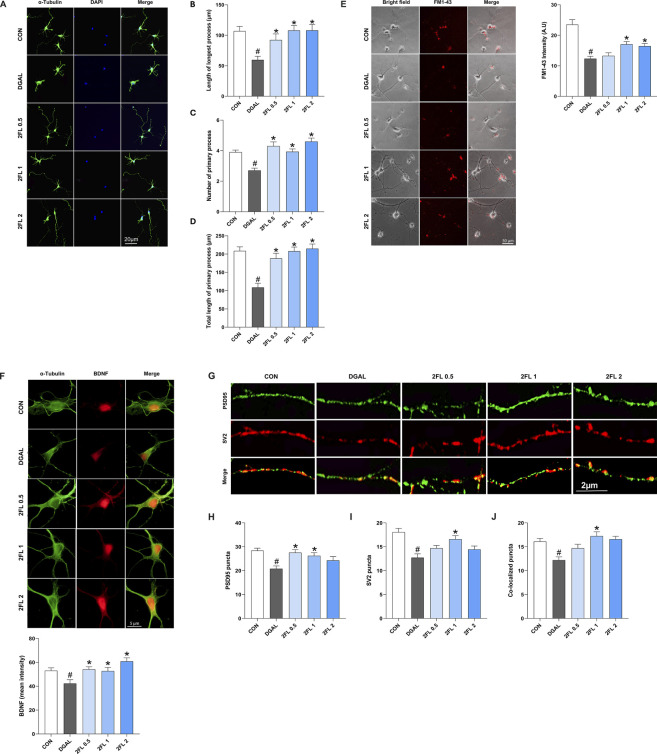
Effect of 2′-FL on synaptic plasticity in hippocampal primary neurons. **(A–D)** Morphometric analysis of neurite outgrowth in DIV3. Fluorescence intensity of **(E)** FM1-43 stained SV accumulation, **(F)** BDNF, and **(G–J)** pre-synaptic protein SV2 and post-synaptic protein PSD95. Data are presented as mean ± SEM (30 neurons per group, and the experiment was independently repeated three times). #p < 0.05 vs. CON, *p < 0.05 vs. DGAL (Panels B, C, D, F, L: Kruskal–Wallis with Dunn’s multiple comparisons test; Panels H, J, K: One-way ANOVA followed by Holm-Sidak’s multiple comparisons test).

The application of 2′-FL, from 0.1 to 2 mg/mL, promoted neuronal growth in a dose-dependent manner (Supplementary Figure S2). Neurons treated with 50 mg/mL d-galactose (108.8 ± 11.61 µm) showed inhibited neurite growth compared to CON (208.7 ± 11.05 µm), whereas treatment with three doses of 2′-FL (0.5 μg/mL, 188.2 ± 14.12; 1 μg/mL, 207.9 ± 11.47; 2 μg/mL, 215 ± 12.49) significantly rescued neurite outgrowth, with the total length of the primary process being 50%, 67%, and 75%, respectively, compared to the model group​ (p < 0.05, Figures 5A–D). The effect of 2′FL on the size of recyclable SV pools in presynaptic terminals was studied using FM1-43 staining. D-galactose (12.36 ± 0.78 A.U) decreased FM1-43 mean intensity compared to CON (23.53 ± 1.59 A.U); however, treatment with 2′-FL at 1 and 2 mg/mL (1 μg/mL, 17.05 ± 0.90; 2 μg/mL, 16.47 ± 0.85) significantly recovered the intensity (p < 0.05, Figure 5E).

To confirm the beneficial effects of 2′-FL on synaptic plasticity, the expression of synaptic markers, including BDNF, postsynaptic density protein-95 (PSD95), and SV protein 2 (SV2), was investigated. D-galactose (42.28 ± 3.18) significantly reduced BDNF expression, which was markedly ameliorated by 2′-FL treatments (0.5 μg/mL, 54.2 ± 2.12; 1 μg/mL, 52.73 ± 2.97; 2 μg/mL, 60.91 ± 2.88) (p < 0.05, Figure 5F). Moreover, 2′-FL 1 μg/mL also reversed the d-galactose-induced decreases in presynaptic marker SV2 (red color) (16.60 ± 0.72) and postsynaptic marker PSD95 (green color) (26.26 ± 1.23), along with their colocalization indicating synapse formation (yellow color) (17.24 ± 0.91) (p < 0.05, Figures 5G–J).

## 4 Discussion

Our current study provides evidence that 2′-FL exerts protective effects on cognitive function, neuroinflammation, oxidative stress, and synaptic plasticity, particularly in neurodegenerative models including AD, using 5xFAD mice and d-galactose-induced neuronal damage. These findings highlights the potential of 2′-FL as a novel therapeutic agent for neurodegenerative conditions.

Behavioral data demonstrated significant improvements in spatial memory and spontaneous alternation in mice treated with 2′-FL. These cognitive benefits may be attributed to the ability of 2′-FL to reduce Aβ accumulation in the hippocampus and cortex of 5xFAD mice. Aβ plaques are well-known drivers of neuroinflammation, as they activate microglia and astrocytes, leading to the release of pro-inflammatory cytokines such as TNF-α and IL-6. Notably, Aβ-induced neuroinflammation involves activation of the NF-κB signaling pathway, a key regulator of inflammatory responses (Fan et al., 2023). Our study showed that 2′-FL inhibited NF-κB activation in microglia, consistent with the observed decrease in pro-inflammatory cytokines in both *in vivo* and *in vitro* models. This suggests that 2′-FL may act upstream by modulating Aβ pathology and suppressing NF-κB activation, thereby alleviating inflammatory responses. Although 2′-FL showed limited effects on tau hyperphosphorylation, its potential influence on tau pathology should not be overlooked. Hyperphosphorylated tau disrupts neuronal structural integrity and is linked to mitochondrial dysfunction, which contributes to excessive reactive oxygen species (ROS) production and oxidative stress (Guo et al., 2020). In this study, 2′-FL effectively mitigated ROS-induced DNA fragmentation and prevented mitochondrial membrane potential (MMP) loss during d-galactose-induced aging in primary hippocampal neurons (PHNs), indicating that 2′-FL acts as a potent free radical scavenger and protects against oxidative damage. These findings highlight its broad role in counteracting tau-induced oxidative stress.

Synaptic plasticity plays a critical role in cognitive function, and its impairment is closely linked to the pathophysiology of neurodegenerative diseases (Zhang et al., 2025). In this study, we observed significant disruptions in synaptic markers, such as BDNF and PSD95 following d-galactose-induced neuronal aging. BDNF is essential for neurotrophic support and the maintenance of synaptic plasticity, while PSD95 is a key scaffolding protein involved in synapse formation, function, and plasticity. Depletion of BDNF and PSD95 contributes to synaptic dysfunction, leading to cognitive decline and memory impairment, as seen in diseases like AD (Dore et al., 2021; Li et al., 2025). Treatment with 2′-FL restored the expression of both BDNF and PSD95, demonstrating its ability to enhance synaptic plasticity and promote neuronal resilience. Additionally, 2′-FL increased expression of the synaptic vesicle marker SV2, which is involved in vesicle recycling, indicating its role in supporting both presynaptic and postsynaptic functions and ultimately improving synaptic connectivity and cognitive performance (Small et al., 2024).

This is the first study to evaluate 2′-FL in the transgenic 5xFAD mouse model, whereas previous studies have primarily used d-galactose-induced aging models (Wang et al., 2022; Gao et al., 2024). Wang et al. (2022) showed that 2′-FL ameliorated oxidative stress and cognitive decline in d-galactose-treated mice by regulating gut microbiota and activating the AMPK/SIRT1/FOXO1 signaling pathway. Similarly, Gao et al. (2024) demonstrated improved learning and memory in APP/PS1 mice treated with 2′-FL, mainly through modulation of gut microbiota. Notably, our study uniquely evaluates the effects of 2′-FL on AD-specific Aβ and tau pathology using the 5xFAD model. We further expanded the mechanistic understanding by examining its impact on synaptic plasticity, neuroinflammation, and oxidative stress in both *in vivo* and *in vitro* systems.

Notably, despite well-documented CNS effects of 2′-FL, no studies have confirmed its presence in the brain or elucidated how it crosses the blood–brain barrier (BBB) (Kuntz et al., 2024). Given the altered BBB permeability observed in neurodegenerative conditions, it is conceivable that 2′-FL may enter the CNS through passive leakage or facilitated transport mechanisms. Although 2′-FL is generally considered impermeable to the BBB under normal physiological states due to its high molecular weight and hydrophilicity, pathological disruption of barrier integrity, such as that seen in AD, may permit the entry of larger or normally excluded molecules. Inflammation-induced tight junction breakdown and endothelial dysfunction could further increase barrier permeability (Sweeney et al., 2018; Sousa et al., 2023). Besides, we do not rule out the possibility that 2′-FL reduces Aβ plaque burden and enhances synaptic plasticity via the gut-brain axis. By reshaping gut microbiota, 2′-FL may elevate circulating gut-derived metabolites that correlate with reductions in Aβ deposition, tau hyperphosphorylation, and neuroinflammatory cytokines (Loh et al., 2024). Furthermore, evidence suggests that 2′-FL improves learning and hippocampal long-term potentiation only when the vagus nerve is intact, implying activation of enteric neurons whose vagal afferents stimulate neurotrophic signaling in the hippocampus to strengthen synaptic connections (Vazquez et al., 2016).

The 5xFAD model harbors five familial AD mutations and more accurately mirrors early AD pathology, including amyloid-beta deposition, synaptic dysfunction, and cognitive decline. However, it shows limited tau tangle formation and does not fully replicate progressive neuronal loss seen in human AD, which may limit its relevance for late-stage disease studies (Lei et al., 2025). In contrast, the d-galactose model does not replicate the full complexity of AD but reliably induces sustained oxidative stress in the hippocampus and cortex, impairs memory and learning, and elevates neuroinflammation and DNA damage markers. This non-genetic model provides a valuable platform for investigating mechanistic pathways and therapeutic interventions targeting age-related neuronal decline (Zhang et al., 2023). Our combined use of both models ensures that conclusions regarding the neuroprotective mechanisms of 2′-FL are robust and relevant across multiple pathways of neurodegeneration, rather than being specific to a single genetic or pathogenic context.

## 5 Conclusion

This study revealed the multifaceted neuroprotective actions of 2′-FL to enhance cognitive function, particularly reducing Aβ accumulation, suppressing neuroinflammation and oxidative stress, and enhancing synaptic plasticity. These findings support the therapeutic potential of 2′-FL in neurodegenerative diseases and cognitive dysfunction. However, further research is warranted to explore its interaction with neuronal receptors and post-translational modifications. This study opens up a promising avenue for the clinical applications of 2′-FL in the context of neuroprotection and cognitive health.

## Data Availability

The original contributions presented in the study are included in the article/Supplementary Material, further inquiries can be directed to the corresponding author.

## Supplementary material

SUPPLEMENTARY FIGURE S1 Effects of d-galactose on neuronal cell viability assessed using the trypan blue exclusion assay.

SUPPLEMENTARY FIGURE S2 Effect of 2′-FL on neuritogenesis.

## Abbreviations

2′-FL, 2′-fucosyllactose; Aβ, amyloid-beta; AD, Alzheimer’s disease; BDNF, brain-derived neurotrophic factor; PHN, primary hippocampal neuron; HPC, hippocampus; IκBα, inhibitor of NF-κB alpha; IL-6, interleukin-6; MMP, mitochondrial membrane potential; NF-κB, nuclear factor kappa-light-chain-enhancer of activated B cells; p-tau, phosphorylated tau; PSD-95, postsynaptic density protein 95; ROS, reactive oxygen species; SV, synaptic vesicle; TNF-α, tumor necrosis factor-alpha; WMT, water maze test.
